# Supplementary material for: Mitochondrial Aldehyde Dehydrogenase 2 Represents a Potential Biomarker of Biochemical Recurrence in Prostate Cancer Patients
Source: Molecules. 2022 Sep 15;27(18):6000. doi: 10.3390/molecules27186000 (PMC9500792; doi:10.3390/molecules27186000)

Supplementary Figure S1. The univariate COX anlaysis of differentially expressed genes in TCGA database.

TCGA-genes	Hazard ratio (95% CI)	P value
CYP4B1	0.921 (0.812-1.045)	0.201
FASN	1.099 (0.863-1.401)	0.444
PTGS2	0.804 (0.700-0.922)	0.002
ACACA	1.123 (0.764-1.652)	0.555
ALDH2	0.756 (0.600-0.953)	0.018
PTGIS	0.877 (0.712-1.079)	0.215
PTGDS	0.855 (0.741-0.987)	0.033
ODC1	1.086 (0.800-1.474)	0.598
UGDH	1.239 (0.905-1.698)	0.182

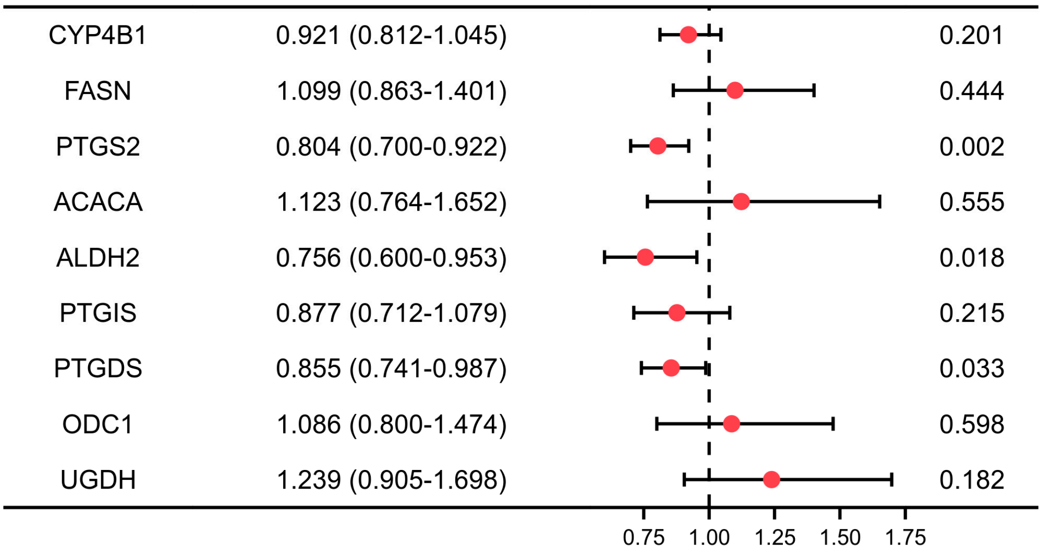

Supplementary Figure S2. The univariate and multivariate COX anlaysis of differentially expressed genes in GEO database.

GEO-genes	Hazard ratio (95% CI)	P value
Univariate analysis		
FASN	0.986 (0.635-1.531)	0.95
ALDH2	0.571 (0.415-0.785)	<0.001
ACACA	0.476 (0.233-0.976)	0.043
CYP4B1	0.749 (0.460-1.218)	0.244
PTGDS	0.834 (0.628-1.109)	0.213
ODC1	1.341 (0.664-2.708)	0.414
UGDH	1.037 (0.809-1.331)	0.773
PTGIS	0.896 (0.586-1.372)	0.615
PTGS2	0.809 (0.526-1.246)	0.336
Multivariate analysis		
ALDH2	0.567 (0.411-0.782)	<0.001
ACACA	0.473 (0.229-0.974)	0.042

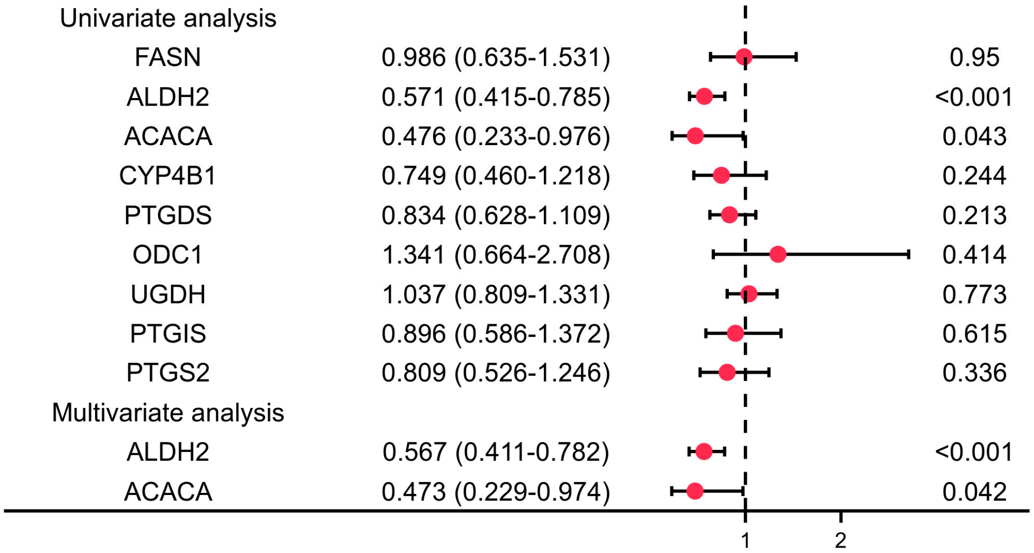

Supplement: Supplementary file 1 [file molecules-27-06000-s001.zip › molecules-1892535-supplementary.pdf]
